# Improved Function After Anterior Controllable Antedisplacement and Fusion for Cervical Ossification of Posterior Longitudinal Ligament: A Long‐Term Follow‐Up

**DOI:** 10.1111/os.14300

**Published:** 2024-11-23

**Authors:** Yangyang Shi, Kaiqiang Sun, Linhui Han, Chen Yan, Jinyu Wang, Jingyun Yang, Yuan Wang, Ximing Xu, Jingchuan Sun, Jiangang Shi

**Affiliations:** ^1^ Department of Orthopedic Surgery, Changzheng Hospital Naval Medical University Shanghai China; ^2^ Rush Alzheimer's Disease Center Rush University Medical Center Chicago Illinois USA; ^3^ Department of Neurological Sciences Rush University Medical Center Chicago Illinois USA

**Keywords:** anterior controllable antedisplacement and fusion, cervical ossification of posterior longitudinal ligament, surgical outcomes

## Abstract

**Background:**

Anterior controllable antedisplacement and fusion (ACAF) is an emerging surgical approach for treating cervical ossification of the posterior longitudinal ligament (C‐OPLL), yet there is limited data on its long‐term efficacy and safety. The present study aimed to analyze the short‐ and long‐term postoperative clinical and radiological outcomes and perioperative complications of ACAF for patients with C‐OPLL.

**Methods:**

This was a single‐center, retrospective, cohort study, with the mean duration of follow‐up of at least 24 months. A total of 111 patients with C‐OPLL in our institution from June 2017 to June 2019 were assessed preoperatively and at 3 days, 3, 6, 12, and 24 months postoperatively. The primary outcome was the recovery of neurological function, measured with the Japanese Orthopedic Association (JOA) score. The secondary outcomes included pain, Cobb angle, spinal canal invasion rate, and surgery‐related complications.

**Results:**

The postoperative JOA score at each follow‐up was significantly better than the preoperative JOA score, regardless of preoperative spinal canal invasion rate, K‐line, and segment length. The visual analog scale (VAS) score also decreased dramatically 3 days after surgery and was maintained at a low level throughout the follow‐up period. Improvements in Cobb angle and invasion rate were observed right after the operation and were maintained for 2 years thereafter.

**Conclusions:**

ACAF could achieve satisfactory recovery of neurological function in C‐OPLL patients during a follow‐up of 24 months, regardless of preoperative spinal canal invasion rate, preoperative K‐line, or surgical segment length.

## Graphical abstract

Instead of resecting the cervical ossification of the posterior longitudinal ligament (C‐OPLL), anterior controllable antedisplacement and fusion (ACAF) achieves ventral decompression by directly moving the vertebrae with OPLL anteriorly, away from the spinal cord. Improvements in Cobb angle and invasion rate were observed right after operation and were maintained for 2 years thereafter. ACAF could achieve satisfactory recovery of neurological function in C‐OPLL patients, regardless of preoperative spinal canal invasion rate, preoperative K‐line, or surgical segment length.

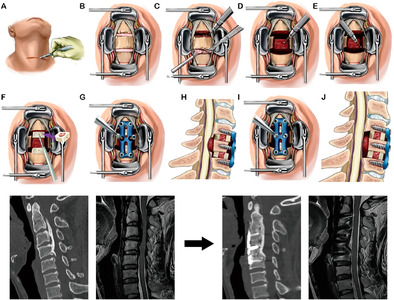

## 1 Introduction

Cervical ossification of the posterior longitudinal ligament (C‐OPLL), characterized by thickening and ossification of the ligaments of the cervical spine, is a chronic and progressive disease and a major cause of cervical myelopathy [1]. Comparatively, East Asians are more susceptible than Caucasians to developing C‐OPLL, with a prevalence of 1.9%–4.3% [2]. Patients with C‐OPLL present with balance or gait instability, loss of fine finger motor control or dexterity, nonspecific upper and lower extremity weakness, paresthesia, pain, and even paralysis. Due to the progressive nature of C‐OPLL, conservative clinical treatment is often ineffective, and surgical treatment has been recommended, especially for patients with progressive neurological deterioration.

Traditional surgical options for the treatment of C‐OPLL can be broadly classified into anterior and posterior approaches. Anterior approaches with or without instrumentation allow ventral decompression of the neural elements by directly resecting the ossified lesion, and satisfactory clinical outcomes have been reported [3, 4]. However, in cases of severe C‐OPLL (i.e., an invasion rate ≥ 60% or involved segments > 3 levels), anterior approaches are technically challenging and can lead to various complications, including cerebrospinal fluid (CSF) leakage, hardware failure, and iatrogenic injury of neural elements [5]. Posterior decompression is indicated in patients with longer segments (3 or more levels) of spinal cord compression or a positive K‐line. It is relatively safer than an anterior approach because it indirectly decompresses the spinal cord without resecting the ventral compression component based on the bowstring effect [6]. However, the extent of decompression of posterior approaches depends on cervical curvature, and the surgical outcomes are unsatisfactory for patients with marked cervical kyphosis [7] or a negative K‐line [8].

Therefore, we proposed a surgical technique, anterior controllable antedisplacement and fusion (ACAF), to treat patients with severe C‐OPLL [9]. Traditional anterior cervical corpectomy and fusion (ACCF) focuses on the excision of an ossified mass, which can cause direct decompression of the spinal cord. Instead of resecting the OPLL, ACAF achieves ventral decompression by directly moving the vertebrae with OPLL anteriorly, away from the spinal cord. Details of the procedure of ACAF were provided in Appendix S1 (Figure S1). Since all the procedures in ACAF are performed outside the spinal canal, CSF leakage, hemorrhage, and intraoperative neural injury can be minimized, making it a safe option for the treatment of severe C‐OPLL. We previously reported that ACAF reduced the occurrence of CSF leakage more than ACCF (3.6% vs. 22.6%) [10]. We also observed from the Japanese Orthopedic Association (JOA) scores that neurological functions improved remarkably in patients who had ACAF compared with patients who had posterior approaches [11]. Although those studies clearly demonstrated the clinical advantages of ACAF over traditional anterior or posterior approaches, they had limited sample sizes and only examined short‐term clinical outcomes. They also did not investigate whether the improvement of neurological functions could be maintained in the long term. Therefore, studies with larger sample sizes and longer durations of follow‐up were required.

Through retrospective analysis of the follow‐up data of ACAF patients with C‐OPLL, this study achieved the following objectives: (i) to investigate both short‐ and long‐term therapeutic effects of ACAF and the impact of factors such as K‐line, segment length, and invasion rate on these outcomes; (ii) to assess radiographic changes in C‐OPLL patients over time following ACAF treatment; (iii) to evaluate the safety profile and incidence of complications associated with ACAF.

## 2 Methods

### 2.1 Patients

In this single‐center study, we reviewed the medical data of patients with C‐OPLL who were treated at the Second Department of Spine Surgery, Changzheng Hospital, from June 2017 to June 2019. OPLL was diagnosed when abnormal calcification occurred longitudinally along the posterior plane of the cervical spine on a lateral computed tomographic scan. All patients met the indications for surgery and were given ACAF at our institution.

Patients were included in the study if they: (a) had symptoms of cervical myelopathy, including limb numbness, impaired motor function, or unsteady gait, due to C‐OPLL; and (b) were treated by ACAF only. Patients were excluded from the study if they: (a) had cervical myelopathy caused by other diseases, including disk herniation or ossification of the ligamentum flavum; (b) had a history of previous cervical surgery; (c) had coexisting cervical diseases, including trauma, infection, and deformity; or (d) had no complete follow‐up data.

This study was approved by the institutional review board of Changzheng Hospital (No. 2020SL059‐2), and all the included patients signed informed consent forms. All the investigators were responsible for the study design and data collection; the corresponding author vouched for the completeness and accuracy of the data and statistical analysis.

### 2.2 Evaluation of Clinical Outcomes

The clinical evaluation was performed by two independent spine surgeons who were unaware of the patients' initial diagnosis. Neurological functions were assessed by the JOA score (range: 0–17, with a higher score indicating better neurological function) [12]. Improve rate (IR) was calculated using the following formula:

$$\mathrm{IR}=\frac{\text{Postoperative}\;\mathrm{JOA}\;\text{score}-\text{Preoperative}\;\mathrm{JOA}\;\text{score}}{17-\text{Preoperative}\;\mathrm{JOA}\;\text{score}}$$

Neck pain was assessed using the visual analog scale (VAS) (range: 0–10, with a higher score indicating more severe neck and arm pain) [13]. Perioperative complications were recorded, including C5 nerve palsy, CSF leakage, dysphagia, and hoarseness. All patients were assessed preoperatively, and then at 3 days, 3, 6, 12, and 24 months postoperatively. Comorbidities, including hypertension and diabetes, were also recorded.

### 2.3 Evaluation of Radiological Outcomes

All patients underwent preoperative and postoperative plain radiography, computed tomography (CT), and magnetic resonance imaging (MRI). In this study, we analyzed the following parameters:

1. Cervical lordosis, measured as the Cobb angle between a line parallel to the posterior aspect of the C2 vertebral body and that of the C7 body [14];
2. K‐line, defined as a straight line that connects the midpoints of the spinal canal at the C2 and C7 levels on the lateral cervical radiographs, including the K‐line positive group (C‐OPLL did not exceed the K‐line) and the K‐line negative group (C‐OPLL exceeded the K‐line) [15];
3. Invasion rate, calculated as the thickness of C‐OPLL at the maximum compressed level divided by the corresponding anteroposterior diameter of the spinal canal on the axial CT images [16].

### 2.4 Statistical Analysis

The primary outcome of interest was neurological function. The secondary outcome of interest included the VAS score, radiological outcomes, and perioperative complications. Continuous data and categorical data were reported as median (interquartile ranges [IQR]) and percentages, respectively. Distribution of the data at baseline and each follow‐up visit was visualized using a combination of box and violin plots. We used the Mann–Whitney *U* test or Kruskal–Wallis one‐way analysis of variance (ANOVA) to test the overall difference and the Dunn test for pairwise comparison between follow‐ups. Multiple comparisons were adjusted using the Holm method to control the family‐wise error rate. To compare functional improvements over time, we adopted nparLD, a popular nonparametric ANOVA‐like test of longitudinal data, which can handle continuous as well as dichotomous, ordinal, and heavily skewed data systematically; it is robust to outliers and exhibits good performance when the sample sizes are small. We also employed ANOVA‐type test statistics (ATS), which is a rank‐based sample approximation from *F*‐distribution's quantiles. We calculated the relative treatment effect (RTE) of each factor (e.g., K‐line), which ranged from 0 to 1. RTE represents the probability that a randomly drawn observation from a particular subgroup (e.g., positive K‐line) had a larger value than that from the whole dataset. An RTE < 0.5 implied a negative effect of the factor, an RTE > 0.5 implied a positive effect, and an RTE = 0.5 implied no effect. In the nparLD analysis, there was no correction of the type I error rate for multiple testing in the pairwise comparison at each follow‐up visit. Consequently, the reported 95% confidence intervals for RTE have not been adjusted for multiplicity and do not imply a definitive effect.

All statistical analyses were performed using *R* version 4.1.0 (https://www.r‐project.org/). The level of statistical significance was set at *p* < 0.05 (two‐sided).

## 3 Results

### 3.1 Basic Characteristics of the Study Participants

A total of 111 patients with C‐OPLL met the eligibility criteria and were included in the evaluation. The median age at surgery was 57 years (IQR: 52–65). The median surgical length was three segments (IQR: 3–4), and about half of the subjects had long surgical segments (> 3 segments). The median preoperative JOA and VAS scores were 12 (IQR: 10–14) and 2 (IQR: 0–3), respectively. The preoperative radiological results showed that 57 patients (51.4%) had a positive K‐line. The median Cobb angle was 13.60° (IQR: 7.35°–20.05°), and the median invasion rate was 54.1% (IQR: 45.1%–62.4%). Detailed information about the study participants is given in Table 1.

**TABLE 1:** Basic characteristic of the study patients.

Characteristics	Value
Sex, female	27 (24.3%)
Age (years, median [IQR])	57.00 (52.00, 65.00)
Height (cm, median [IQR])	170.00 (162.50, 172.50)
Weight (kg, median [IQR])	72.00 (62.50, 80.50)
Surgical length (median [IQR])	3.00 (3.00, 4.00)
Surgery segment	
Long (> 3 segments)	55
Short (≤ 3 segments)	56
Pre‐clinical scores	
JOA (median [IQR])	12.00 (10.00, 14.00)
VAS (median [IQR])	2.00 (0.00, 3.00)
Pre‐radiological evaluation	
K‐line, positive	57 (51.4%)
Cobb angle (°, median [IQR])	13.60 (7.35, 20.05)
Occupying rate (%, median [IQR])	54.11 (45.06, 62.42)

Abbreviations: JOA, Japanese Orthopedic Association; VAS, Visual Analogue Scale.

### 3.2 Neurological Functions

**FIGURE 1:**
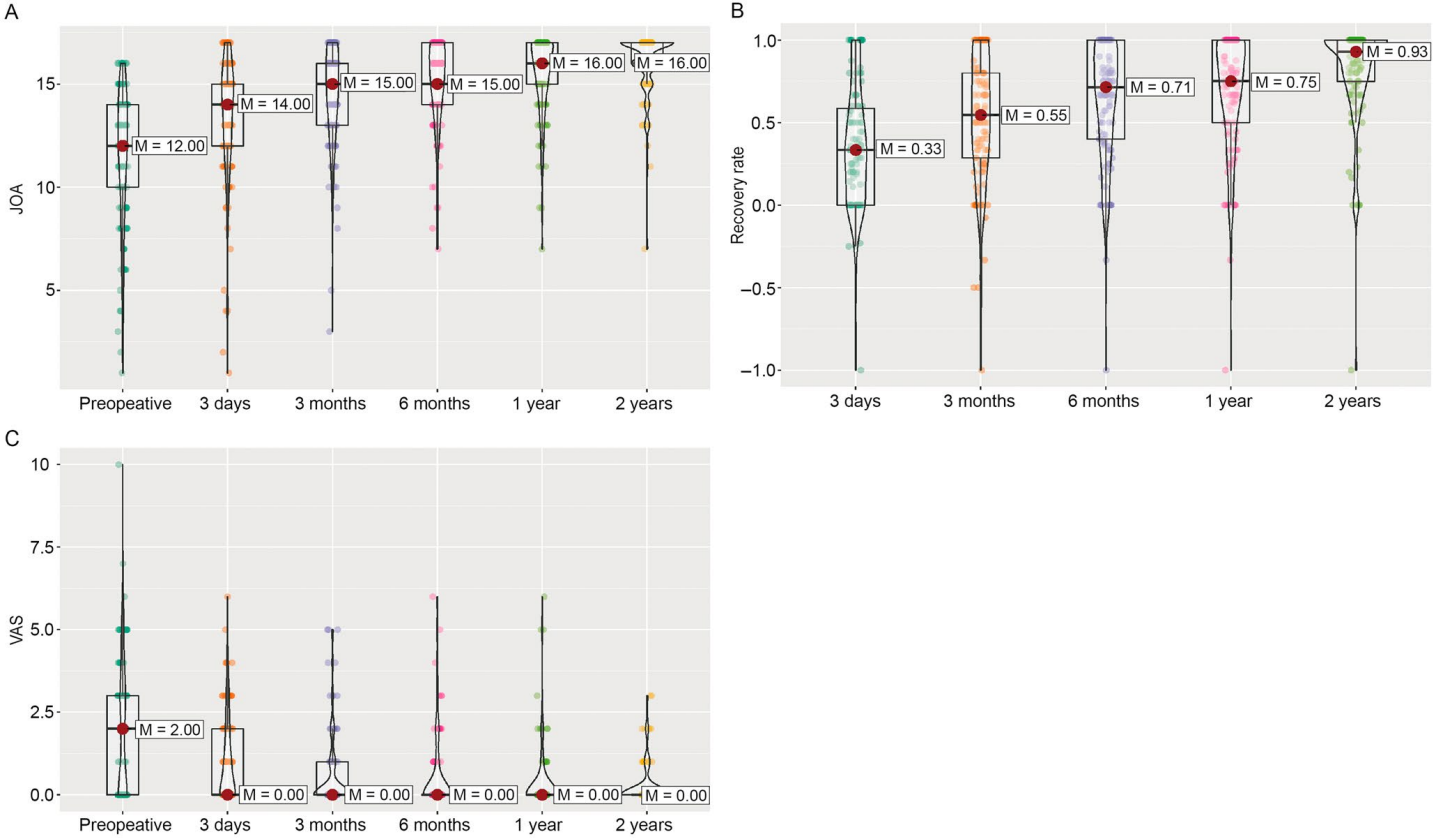
Neurological function of the patients before and after surgery. (A) JOA score during the follow‐up. (B) JOA recovery rate during the follow‐up. (C) VAS score during the follow‐up. JOA, Japanese Orthopedic Association; M, median; VAS, Visual Analogue Scale.

The postoperative JOA score at each follow‐up was significantly better than the preoperative JOA score (each *p* ≤ 0.001) (Figure 1A). We observed an overall gradual improvement in postoperative JOA score, with a peak at 3 months, indicating that ACAF was effective in improving neurological functions, even at the early stage after the operation. This continuous improvement of neurological function could be maintained for 2 years after the surgery.

**FIGURE 2:**
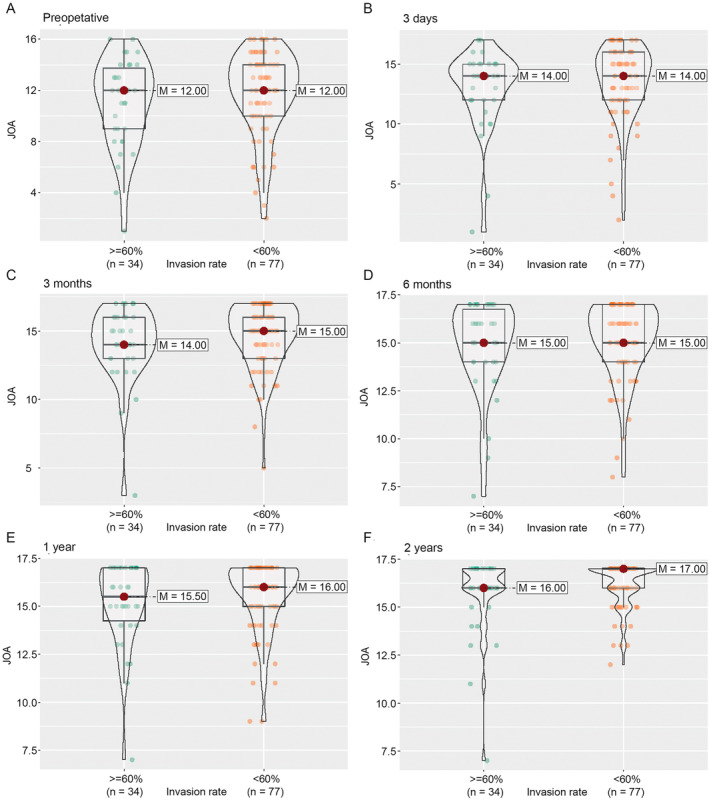
The distribution of JOA score by preoperative spinal canal invasion rate during the follow‐up. (A) Preoperative. (B) Three days after operation. (C) Three months after operation. (D) Six months after operation. (E) One year after operation. (F) Two years after operation. JOA, Japanese Orthopedic Association; M, median.

**FIGURE 3:**
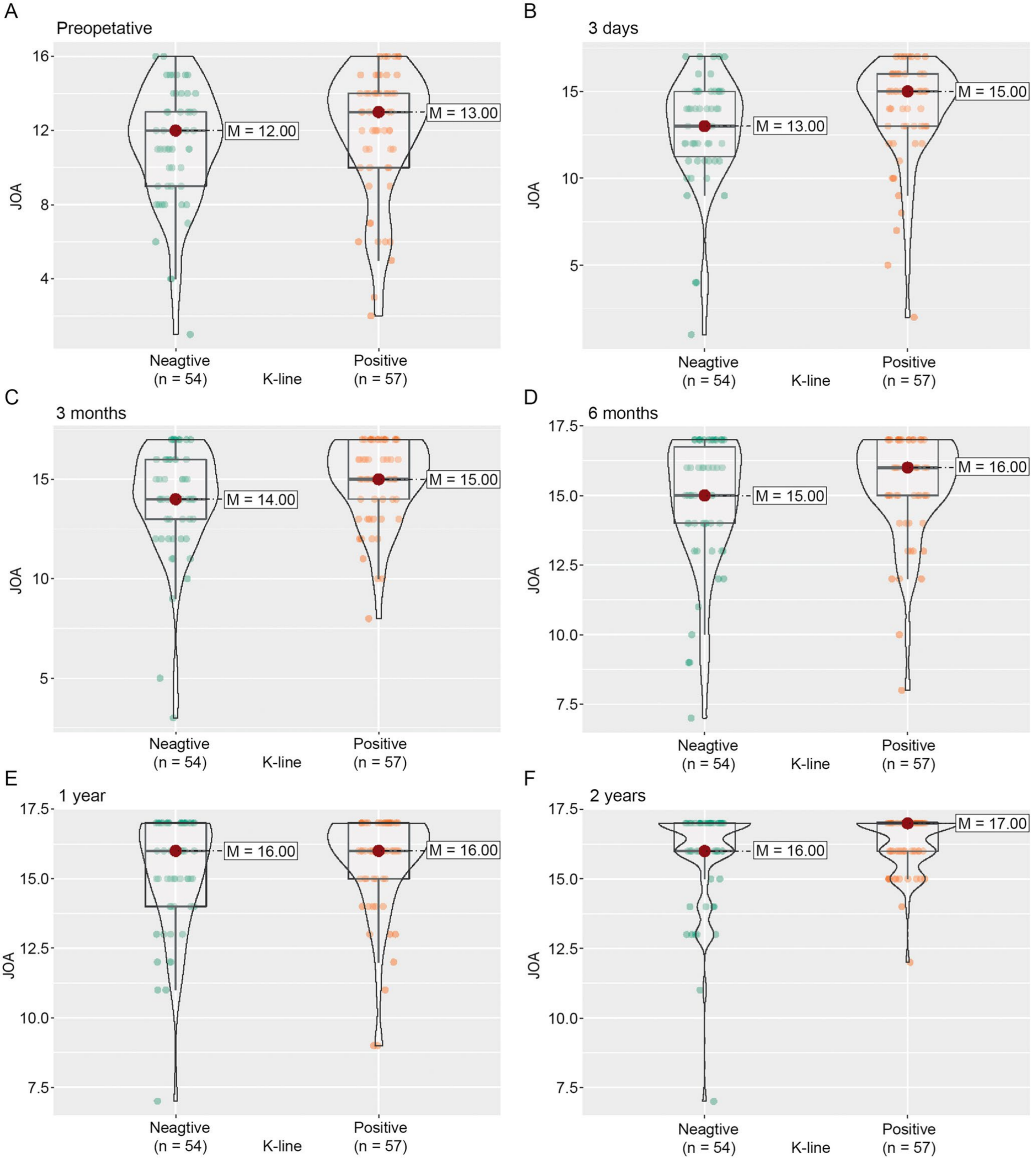
The distribution of JOA score by preoperative K‐line during the follow‐up. (A) Preoperative. (B) Three days after operation. (C) Three months after operation. (D) Six months after operation. (E) One year after operation. (F) Two years after operation. JOA, Japanese Orthopedic Association; M, median.

**FIGURE 4:**
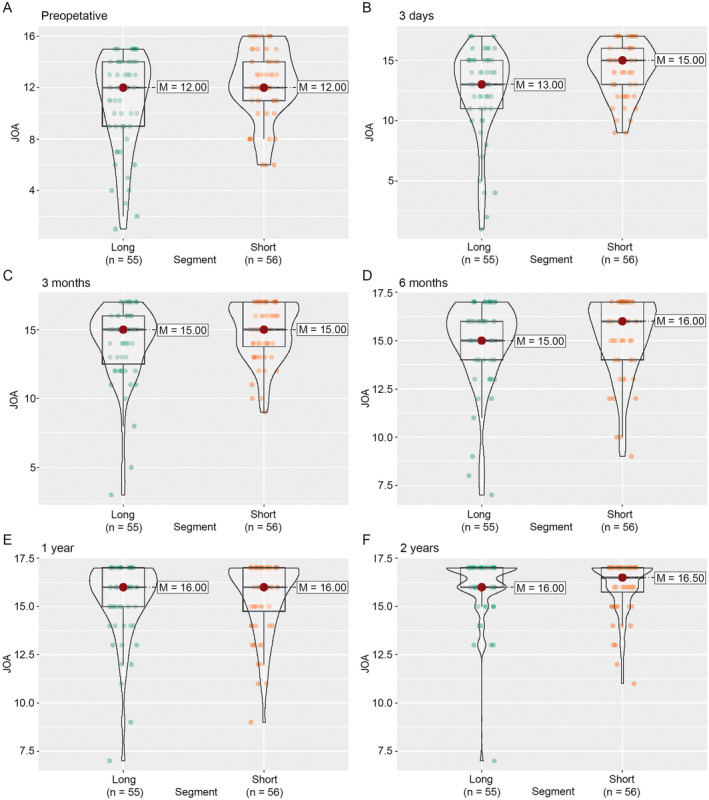
The distribution of JOA score by surgical length during the follow‐up. (A) Preoperative. (B) Three days after operation. (C) Three months after operation. (D) Six months after operation. (E) One year after operation. (F) Two years after operation. Short segments: ≤ 3 segments, and long segments:> 3 segments. JOA, Japanese Orthopedic Association; M, median.

Using nparLD, we examined the effects of preoperative spinal canal invasion rate (≥ 60% vs. < 60%), preoperative K‐line, and surgical segment length, on JOA scores over time. We found no significant overall effect of the preoperative spinal canal invasion rate on the JOA score (RTE = 0.47 and 0.51 for greater and smaller preoperative spinal canal invasion rates, respectively; *p* = 0.06 from ATS). There was no significant difference in JOA score between the two groups before surgery and at each follow‐up after surgery (Figure 2). Similarly, we found no significant overall effect of K‐line (RTE = 0.54 and 0.46 for positive and negative K‐line, respectively; *p* = 0.29 from ATS) and segment length (RTE = 0.47 and 0.53 for long and short segment, respectively; *p* = 0.12 from ATS). There was no significant difference in JOA scores between the two respective groups before surgery and at each follow‐up after surgery, except for a difference in preoperative and Day 3 postoperative JOA scores between those who had a long segment and those who had a short segment (Figures 3 and 4). The individual follow‐up information on the effect of preoperative spinal canal invasion rate, K‐line, and segment length on JOA scores are given in Figure S2.

We observed that patients' recovery rates also gradually increased during the follow‐up period (Figure 1B), indicating that patients with C‐OPLL benefited from ACAF in their recovery rate during that time. We found no significant overall effect of preoperative spinal canal invasion rate (RTE = 0.49 and 0.50 for high invasion (≥ 60%) and low invasion (< 60%) rates, respectively, *p* = 0.53 from ATS); of K‐line (RTE = 0.49 each for both negative and positive K‐line, *p* = 0.795 from ATS); or of segment length (RTE = 0.49 and 0.51 for long and short segment length, respectively, *p* = 0.63 from ATS). The individual follow‐up information for preoperative spinal canal invasion rate, preoperative K‐line, and segment length on the recovery rate are given in Figure S3.

We also observed that patients' pain symptoms improved significantly after ACAF. The VAS score decreased at Day 3 after operation, compared with the preoperative level, and was maintained at a low level during the whole follow‐up (Figure 1C), indicating that ACAF can alleviate pain symptoms in patients with C‐OPLL. We found no significant effect of preoperative spinal canal invasion rate (RTE = 0.52 and 0.49 for greater and smaller invasion rates, respectively; *p* = 0.517 from ATS); of K‐line (RTE = 0.50 and 0.50 for positive and negative K‐line, respectively; *p* = 0.995 from ATS). The individual follow‐up information for preoperative spinal canal invasion rates and preoperative K‐line on patients' pain symptoms are given in Figure S4A,B, respectively. However, the nparLD analysis indicated a significant effect of segment length (RTE = 0.47 and 0.54 for long and short segment lengths, respectively; *p* = 0.03 from ATS; the individual follow‐up is given in Figure S4C). During the entire follow‐up period, patients with short segments tended to have higher VAS scores. However, the Mann–Whitney *U* analysis showed no significant difference in VAS score at any point during the follow‐up after correction for multiple testing (Figure S5). These results indicated that ACAF alleviated pain‐related symptoms in patients with C‐OPLL regardless of different preoperative spinal canal invasion rates, preoperative K‐line, and surgical segments.

### 3.3 Radiological Outcomes

**FIGURE 5:**
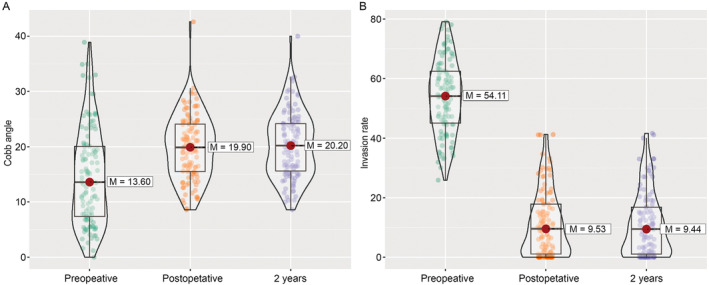
Radiological parameters of the patients before and after surgery. (A) The distribution of Cobb angle during the follow‐up. (B) The distribution of spinal canal invasion rate during the follow‐up. M, median.

Postoperative Cobb angles improved significantly over time, compared with the preoperative level (*p* ≤ 0.001 from ATS). Specifically, the Cobb angle increased (median: 19.90° [IQR: 15.50°–24.10°] vs. 13.60° [IQR: 7.35°–20.05°], *p* ≤ 0.001), and was maintained at a high level 2 years after operation (median: 20.20° [IQR: 15.60°–24.20°] vs. 13.60° [IQR: 7.35°–20.05°], *p* ≤ 0.001) (Figure 5A). The postoperative spinal canal invasion rate also improved significantly over time, compared with the preoperative level (*p* ≤ 0.001 from ATS). Specifically, the invasion rate decreased after operation (median: 9.5% [IQR: 1.1%–17.8%] vs. 54.11% [IQR: 45.1%–62.4%], *p* ≤ 0.001), and was maintained at a low level 2 years after the operation (median: 9.4% [IQR: 1.0%–16.8%] vs. 54.1% [IQR: 45.1%–62.4%], *p* ≤ 0.001) (Figure 5B).

### 3.4 Surgery‐Related Complications

Surgery‐related complications were rare in general (Table 2). The most prevalent complication was dysphagia (9.1%), followed by CSF leakage (2.7%) and hoarseness (2.7%), indicating that the complications of ACAF are few and moderate, making it a relatively safe surgical technique. No vertebral artery injuries and fusion‐related issues were encountered in these 111 patients.

**TABLE 2:** Surgery‐related complications during the follow‐up period.

Complications (*n*/%)	Value
C5 nerve palsy	2 (1.8%)
Cerebrospinal fluid leakage	4 (3.6%)
Dysphagia	10 (9.1%)
Hoarseness	3 (2.7%)
Total	19 (17.1%)

### 3.5 Case Report

**FIGURE 6:**
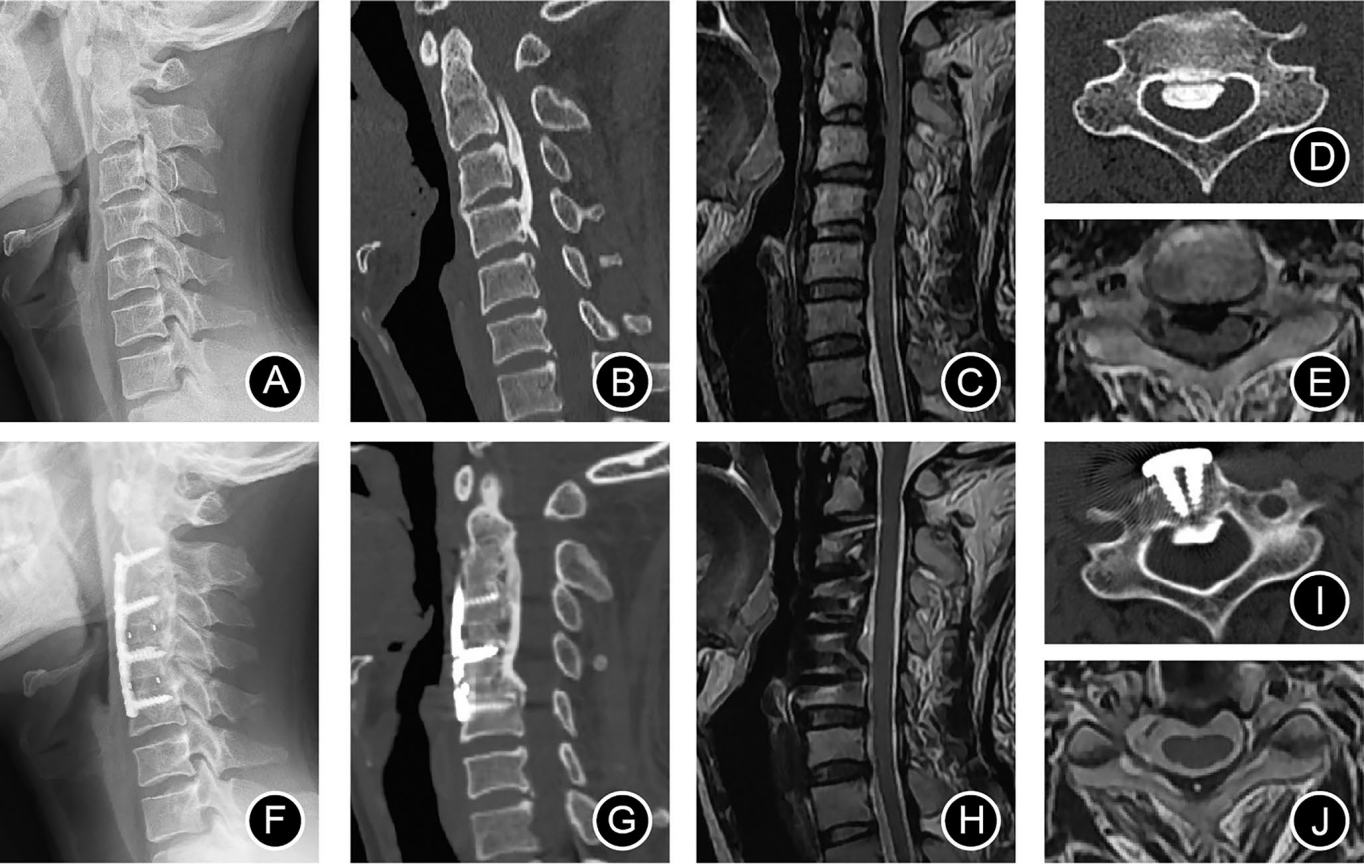
Imaging of a 32‐year‐old man undergoing ACAF. (A) Lateral X‐ray showing the cervical lordosis was 5°. (B) Sagittal CT of the cervical spine showing spine stenosis and severe OPLL from C2 to C4/5. (C) Sagittal MRI showing spinal cord compression from C2 to C4/5. (D) Cross‐sectional CT showing the invasion rate of 60.3%. (E) Cross‐sectional MRI showing the morphology of the spinal cord was crescent. (F) Lateral X‐ray showing a surgery of ACAF from C2 to C5 and the cervical lordosis was 13°. (G) Sagittal CT and (H) MRI showing sufficient decompression and the CSF band reappear at operating level. (I) Cross‐sectional CT showing the invasion rate of 24.5%. (J) Cross‐sectional MRI showing the morphology of the spine cord returning to normal. Instead of resecting the cervical ossification of the posterior longitudinal ligament (C‐OPLL), anterior controllable antedisplacement and fusion (ACAF) achieve ventral decompression by directly moving the vertebrae with OPLL anteriorly, away from the spinal cord. Improvements in Cobb angle and invasion rate were observed righ the after operation and were maintained for 2 years thereafter. ACAF could achieve satisfactory recovery of neurological function in C‐OPLL patients, regardless of preoperative spinal canal invasion rate, preoperative K‐line, or surgical segment length.

A 32‐year‐old man presented with complaints of severe neck pain, and an inability to walk at a brisk pace along with a feeling of heaviness and weakness in right leg. The symptoms had aggravated 1 month before admission. As shown in Figure 6, ACAF was performed from C2 to C5. The cervical lordosis was corrected from 5° preoperatively to 13° postoperatively. The invasion rate decreased from 60.3% preoperatively to 24.5% postoperatively. After the operation, the patient showed dramatical recovery of neurological function. The JOA score increased from 9 to 15. There were no complications during follow‐up.

## 4 Discussion

This retrospective evaluation reveals that all patients with C‐OPLL who underwent ACAF achieved satisfactory recovery of neurological function 2 years after surgery (median JOA score: 16; median recovery rate: 93%), without regard to preoperative spinal canal invasion rate, preoperative K‐line, or surgical segments. The larger sample size and longer follow‐up highlights that ACAF could be a reliable and effective alternative treatment for C‐OPLL patients with different preoperative spinal canal invasion rates, different K‐lines, and different surgical segments. The results for the secondary outcomes, including pain, Cobb angle, spinal canal invasion rate, and surgery‐related complications, are like those of the primary outcomes.

### 4.1 Impact of Spinal Canal Invasion Rate on Neurological Recovery With ACAF

In this study, the JOA score was the primary indicator of patients' neurological function [17, 18]. There was a gradual improvement in the patients' postoperative JOA scores throughout the follow‐up, with a median JOA score of 16 (mean: 15.33 ± 1.97) at 12 months after surgery. This is in contrast to the observation in a previous study of a JOA score of 14.68 ± 2.87 in patients with C‐OPLL at 12‐month follow‐up [19]. In our study, the patients with severe C‐OPLL (invasion rate ≥ 60%) had a median JOA score of 16.0 (mean: 15.99 ± 1.52) at 24‐month follow‐up. In contrast, an extant prospective nonrandomized controlled study reported that in patients with severe C‐OPLL, the mean JOA score at 24‐month follow‐up was 14.0 ± 2.1 and 15.2 ± 2.8 for the anterior and posterior approaches, respectively [20]. Another study also reported that at 24 months, the JOA scores of patients with severe C‐OPLL were 14.7 ± 1.9 and 14.2 ± 1.6 for the anterior and posterior approaches, respectively [21]. These results indicate that ACAF produced better neurological functions in the treatment of C‐OPLL, whether it was severe or not. All patients in our study had a median recovery rate of 93% at 24 months, while the median recovery rate was 88% in those with severe C‐OPLL. However, a previous study reported that in patients with severe C‐OPLL, the recovery rate at 24 months postoperative follow‐up was 61.6% for ACCF and 55.8% for posterior approaches. Furthermore, a recent network meta‐analysis comparing the three different surgeries for C‐OPLL concluded that ACAF was the best for improving recovery rate, followed by ACCF and posterior approaches [22]. Our findings demonstrate the advantage of ACAF in improving and maintaining neurological functions in the long term.

### 4.2 Impact of Surgical Segment Length on Neurological Recovery With ACAF

Multilevel C‐OPLL (long segment > 3 levels) is often encountered in clinical practice. The anterior approach has been more effective for multilevel C‐OPLL compared with posterior laminoplasty due to its direct removal of the ossified mass [23]. A previous study with a follow‐up of 48 months found that ACCF achieved better neurological function than laminoplasty (JOA score: 14.2 ± 1.3 vs. 12.4 ± 1.2; recovery rate: 63.2% ± 15.2% vs. 43.5% ± 12.7%) [24]. However, the incidence of CSF leakage after ACCF in multilevel C‐OPLL is relatively high (11.4%–18.2%) [24, 25]. The posterior approach, conversely, is safer and less technically demanding [26]. However, its technique depends largely on invasion rate and cervical lordosis. Therefore, an optimal technique should combine the advantages of the anterior approach (i.e., directly decompressing the spinal cord) and the safety of the posterior approach, without regard to the invasion rate or cervical curvature. This study shows that ACAF is a potential optimal technique for the treatment of multilevel C‐OPLL. Although the patients with a long segment showed relatively lower JOA scores than those with a short segment, 3 days after surgery (Figure 4B; *p* = 0.012), no significant differences were observed in the JOA score between the two groups at 3, 6, 12, and 24 months after surgery (Figure 4C–F; all *p* > 0.05). The changes in recovery rate were also like those of the JOA score. However, the recovery of neurological function (JOA scores and recovery rate) of patients 24 months after surgery in this study was better than that of patients treated by ACCF [24, 25]. We observed that 5.5% (3/55) of patients with long segment C‐OPLL experienced CSF leakage after ACAF, which might be related to over‐antedisplacement of the ossified mass. Nevertheless, the leakage was easily controlled by plugging the bilateral bone grooves in a procedure with minimal dural defect. Collectively, the results in this study demonstrate that ACAF can be an effective and safe option for treating patients with C‐OPLL, regardless of the segment length involved.

### 4.3 Impact of K‐Line on Neurological Recovery With ACAF

K‐line is an important factor in the neurological function of patients with C‐OPLL [27]. Previous research has reported that a posterior approach should not be recommended for patients with a negative K‐line due to an unfavorable recovery rate [28]. ACCF has been frequently recommended for patients with a negative K‐line. However, this technique is technically demanding, with a higher incidence of complications. In the present study, the patients with negative K‐lines had similar neurological recoveries, as shown by their JOA scores and recovery rates at each follow‐up, to those with positive K‐lines (Figure 3). Previous studies reported that at 12 months after surgery, the recovery rate was 65.2% and 20.7% for patients with negative K‐lines treated by ACCF and posterior laminoplasty, respectively [29]. By contrast, we found a median recovery rate of 75% with ACAF at 12 months after surgery, which is much higher than other approaches [28, 29]. The recovery rate increased to 89% at 24 months after surgery. Further, nparLD analysis revealed that K‐line had no effect on ACAF for treating patients with C‐OPLL. The results suggest that patients with C‐OPLL can be treated by ACAF, regardless of K‐line.

### 4.4 Radiographic Outcomes of ACAF

From the radiological evaluations, the Cobb angle of patients improved after surgery and was maintained during the entire 24‐month follow‐up, which indicates that the clinical effect of ACAF on cervical stability was reliable (Figure 5A). ACAF also decreased the invasion rate of the spinal canal compared with the preoperation level (9.53% vs. 54.11%, *p* < 0.05). Moreover, the median invasion rate at the final 24 months of follow‐up did not increase compared with that at 12 months after surgery, suggesting that there was no progression of C‐OPLL after ACAF, at least within the 24 months of follow‐up. We attribute this result to the stable cervical construction and non‐manipulation imposed on the posterior cervical structure after ACAF, since cervical instability is a risk factor for the progression of C‐OPLL. However, a longer follow‐up is still required to validate the results of this study.

### 4.5 Safety of ACAF

CSF leakage is a common complication of anterior decompression surgery for OPLL, with reported incidence rates ranging from 4% to 32%, averaging 8.3% in patients undergoing ACCF [30]. However, the results of our study indicated that the incidence of CSF leakage after ACAF was only 1.8%, significantly lower than the incidence following traditional anterior cervical surgery. This lower rate is attributed to the technique in ACAF, where the vertebral ossification complex (VOC) is shifted anteriorly instead of being directly removed, minimizing the risk of CSF leakage by reducing the disruption of the ossification–dural interface. Furthermore, the incidence of C5 paralysis in ACAF was significantly lower than in posterior surgery [14], as it involves controlled lifting of the vertebrae–OPLL complex and simultaneous bilateral decompression, which prevents excessive spinal cord movement. Although ACAF is associated with a higher incidence of early postoperative complications such as dysphagia and hoarseness due to esophageal traction, these complications typically alleviate gradually without requiring specific treatment.

## 5 Limitations and Strengths

This study has several limitations that should be acknowledged. First, as a retrospective study without randomization, there was an inherent selection bias in the choice of surgical procedures. Second, neurological status was assessed using the JOA scoring system, which does not incorporate patient‐reported evaluations. Third, our study did not have a control group. In fact, the objective of this present study was to elucidate the effects of ACAF on patients to reveal its long‐term surgical benefits. It is certainly helpful to compare ACAF with other surgical techniques. We are conducting a multicenter, randomized, open‐label, parallel‐group, active‐controlled trial that will compare the clinical benefits of ACAF versus conventional posterior laminoplasty (LAMP) in severe COPLL patients. We will publish the results after the completion of data collection.

Despite these limitations, our study has the following advantages: (a) our research included a substantial number of patients, allowing for greater statistical power and more reliable findings; (b) the follow‐up was conducted for a minimum duration of 24 months. This extended period enables us to capture the full scope of clinical outcomes and potential long‐term effects, providing a more comprehensive understanding of ACAF's efficacy and safety over time; (c) a more rigorous analytical approach, nparLD, was adopted, which can handle different types of longitudinal data and is robust to data distribution and outliers, leading to more accurate estimates.

## 6 Conclusion

This present study suggests that ACAF could achieve satisfactory recovery of neurological outcomes in C‐OPLL patients during a follow‐up of 24 months, regardless of preoperative spinal canal invasion rate, preoperative K‐line, and surgical segment length. The results highlight that ACAF could be a reliable and effective alternative in treating C‐OPLL patients, with wider surgical indications.

## Author Contributions

**Yangyang Shi:** conceptualization, writing – original draft, investigation. **Kaiqiang Sun:** conceptualization, investigation, writing – review and editing. **Linhui Han:** data curation, writing – review and editing. **Chen Yan** and **Jinyu Wang:** formal analysis, methodology. **Jingyun Yang** and **Yuan Wang:** visualization, software. **Ximing Xu:** supervision, visualization. **Jingchuan Sun:** methodology. **Jiangang Shi:** conceptualization, project administration, writing – review and editing.

## Disclosure

All authors listed meet the authorship criteria according to the latest guidelines of the International Committee of Medical Journal Editors, and all authors are in agreement with the manuscript.

## Ethics Statement

All procedures performed in studies involving human participants were in accordance with the ethical standards of the Ethics Committee of Changzheng Hospital.

## Conflicts of Interest

The authors declare no conflicts of interest.

## Supporting information


**Appendix S1.** Supporting Information.
