# Supplementary material for: Improved Function After Anterior Controllable Antedisplacement and Fusion for Cervical Ossification of Posterior Longitudinal Ligament: A Long‐Term Follow‐Up
Source: Orthop Surg. 2024 Nov 23;17(2):416–26. doi: 10.1111/os.14300 (PMC11787986; doi:10.1111/os.14300)
Supplement: Supplementary file 1 — Appendix S1. Supporting Information. [file OS-17-416-s001.docx]

**Supplementary Appendix**

**Table of Contents**

| **Supplemental Figures** | **Page no.**  **(Suppl.)** |
| --- | --- |
| Figure S1: Illustration of the surgical procedure of ACAF. | 2-3 |
| Figure S2: Relative effect of preoperative risk factors on JOA score. | 4 |
| Figure S3: Relative effect of preoperative risk factors on recovery rate. | 5 |
| Figure S4: Relative effect of preoperative risk factors on VAS. | 6 |
| Figure S5: The distribution of VAS score by surgical length during the follow-up. | 7 |

**Surgical procedure of ACAF**

The detailed procedure of ACAF, illustrated in Figure 1, has been reported in previous studies.^13-16^ However, as an overview, under general endotracheal anesthesia, with the patient in a supine position, a surgical incision for a right-sided Smith-Robinson approach was made (Figure S1A). After exposure of the anterior surface of the vertebral body, disks at the involved levels were resected (Figures S1B and S1C), exposing the posterior longitudinal ligament (PLL). The anterior parts of the involved vertebral bodies were then partially resected at the level of the thickest ossified mass. (Figure S1D). Bilateral bony troughs were made while preserving the base of the ipsilateral bony trough (Figure S1E). Then, the intervertebral cages filled with autogenic bone were inserted at the corresponding intervertebral space (Figure S1F). An appropriately pre-bent plate and screws were placed to stabilize the complex temporarily, followed by the resection of the base of the ipsilateral bony trough to complete the isolation of the vertebrae–C-OPLL complex from the surrounding bony structures (Figures S1G and S1H). The complex was antedisplaced anteriorly by gradually tightening the screws in each vertebra at the same pace (Figures S1I and S1J). On completion, an autogenous bone graft or allograft was placed in the bilateral longitudinal bony troughs for fusion. Finally, the incision was sutured in layers, and a neck brace was fitted and routinely used for 3 months.


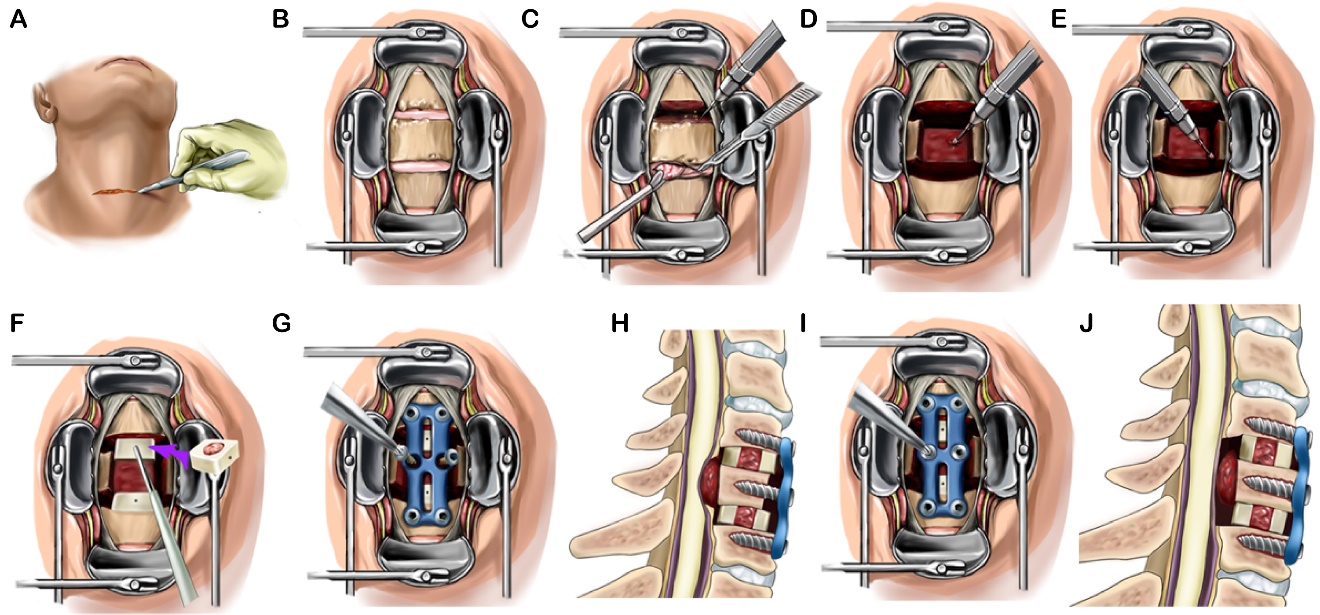


**Figure S1. Illustration of the surgical procedure of ACAF**.

A) Establishment of right-sided Smith-Robinson incision; B) Exposure of anterior surface of vertebral body; C) Removal of involved discs. D) Removal of the anterior portion of the involving vertebral bodies and. E) Establishment of bilateral bony troughs; F) Insertion of intervertebral cages; G and H) Prefixation of the cervical plate and screws; and I and J) Controllable antedisplacement of the complex.

ACAF: anterior controllable antedisplacement and fusion.

**Figure S2. Relative effect of preoperative risk factors on JOA score.**


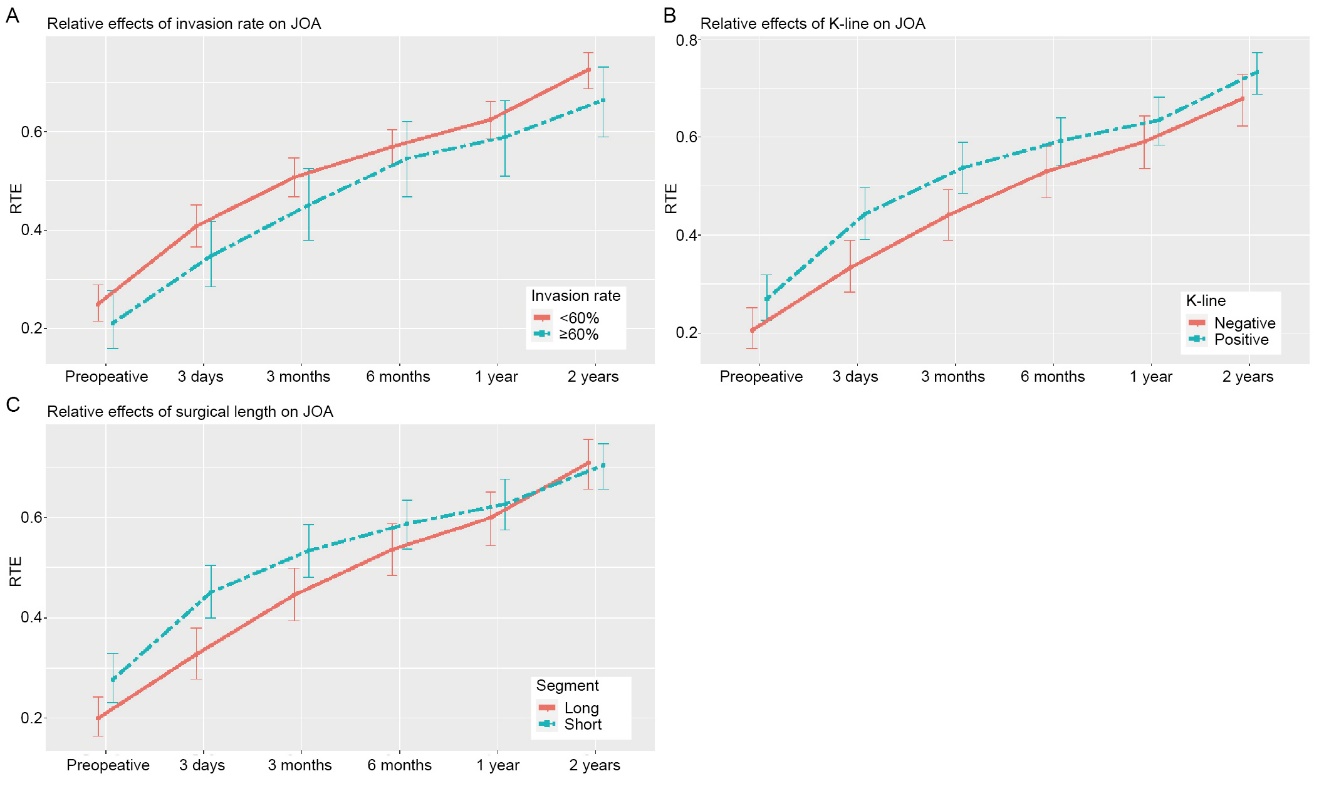


A) Spinal canal invasion rate; B) K-line; and C) Surgical length.

JOA, Japanese Orthopedic Association; RTE, relative treatment effect.

Short segments: ≤ 3 segments; long segments: ＞3 segments.

**Figure S3. Relative effect of preoperative risk factors on recovery rate.**


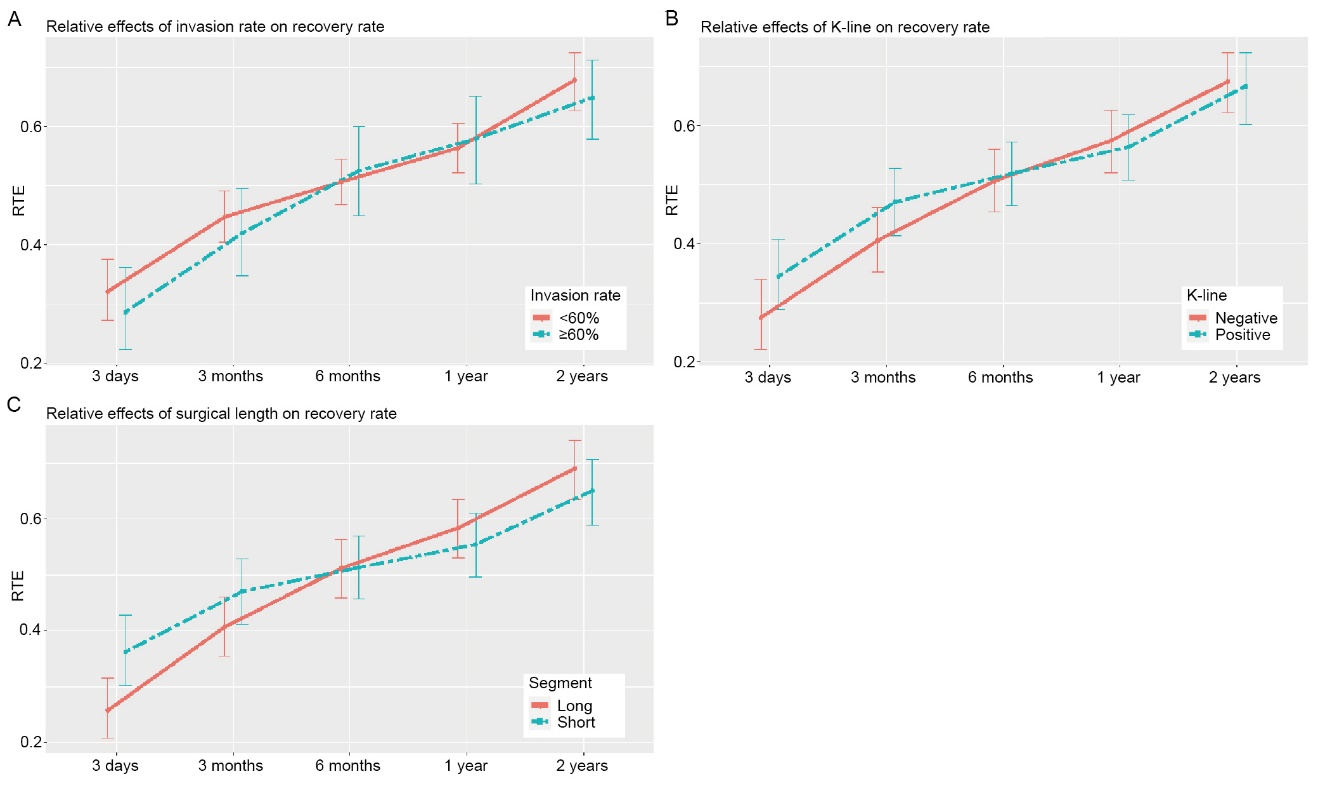


A) Spinal canal invasion rate; B) K-line; and C) Surgical length.

Short segments: ≤ 3 segments; long segments: ＞3 segments**.**

RTE, relative treatment effect.

**Figure S4. Relative effect of preoperative risk factors on VAS.**


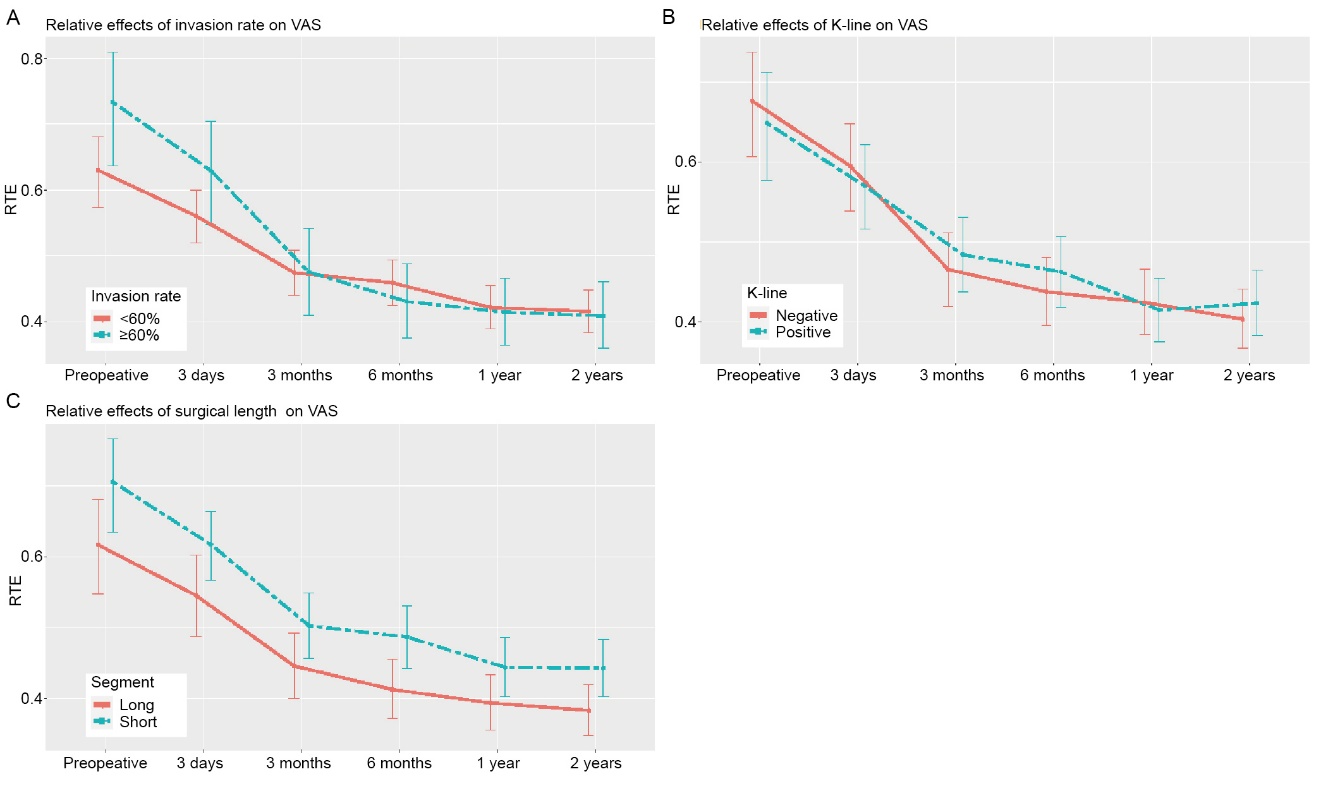


A) Spinal canal invasion rate; B) K-line; and C) Surgical length.

Short segments: ≤ 3 segments), and long segments: ＞3 segments.

RTE, relative treatment effect; VAS, Visual Analogue Scale.

**Figure S5. The distribution of VAS score by surgical length during the follow-up.**


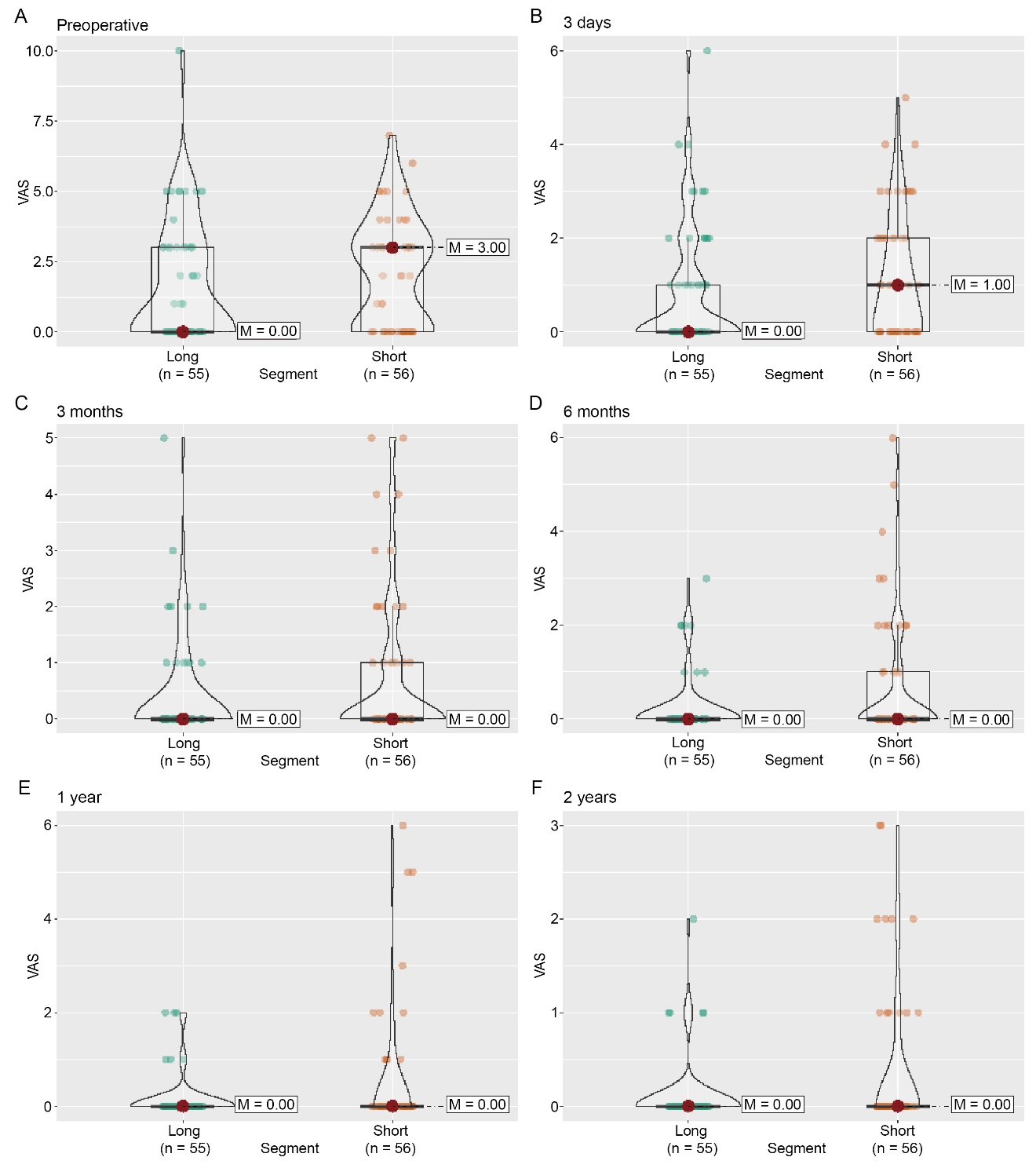


A) Preoperative; B) Three days after operation; C) Three months after operation; D) Six months after operation; E) One year after operation; and F) Two years after operation.

Short segments: ≤ 3 segments), and long segments: ＞3 segments.

VAS, Visual Analogue Scale; M, median.
